# Multi-Electrode Alpha tACS During Varying Background Tasks Fails to Modulate Subsequent Alpha Power

**DOI:** 10.3389/fnins.2018.00428

**Published:** 2018-06-25

**Authors:** Tomer Fekete, Andrey R. Nikolaev, Floris De Knijf, Aleksandra Zharikova, Cees van Leeuwen

**Affiliations:** ^1^Brain and Cognition Research Unit, Laboratory for Perceptual Dynamics, KU Leuven, Leuven, Belgium; ^2^Academic Center for General Practice, KU Leuven, Leuven, Belgium

**Keywords:** transcranial current stimulation, EEG, alpha rhythm, multi electrode stimulation, alternating

## Abstract

Transcranial alternating-current stimulation (tACS) for entraining alpha activity holds potential for influencing mental function, both in laboratory and clinical settings. While initial results of alpha entrainment are promising, questions remain regarding its translational potential—namely if tACS alpha entrainment is sufficiently robust to context and to what extent it can be upscaled to multi-electrode arrangements needed to direct currents into precise brain loci. We set out to explore these questions by administering alternating current through a multi-electrode montage (mtACS), while varying background task. A multi-electrode analog of previously employed anterior/posterior stimulation failed to replicate the reported alpha entrainment, suggesting that further work is required to understand the scope of applicability of tACS alpha entrainment.

## Introduction

A wide range of functions, from tonic inhibition (arousal) to selective attention and adaptive control, have been associated with the cortical alpha rhythm (Sadaghiani and Kleinschmidt, 2016). However, in order to go beyond mere correlations and establish the causal role of alpha oscillations, the expression of such activity must be brought under experimental control. *In vivo* studies have shown that a powerful tool for enhancing intrinsic network rhythmic activity is entrainment through sinusoidal stimulation (Fröhlich and McCormick, 2010). This suggests that effectuating entrainment of the alpha rhythm might be achieved through transcranial stimulation methodologies. Initial results using either transcranial alternating current stimulation (tACS) or transcranial magnetic stimulation (TMS) offer some support for the viability of this approach (Romei et al., 2010; Zaehle et al., 2010; Neuling et al., 2013; Helfrich et al., 2014a).

However, concerns can be raised as to the translational potential of alpha entrainment through tACS: first, Neuling et al. (2013) found that tACS could entrain alpha when eyes are open, but not when they are shut (a finding later indirectly confirmed by Ruhnau et al., 2016). As shutting the eyes induces strong spontaneous alpha oscillations, this suggests the existence of a ceiling effect—perhaps alpha can be coaxed to increase only up to some maximal level (at least within the range of tACS intensity that does not elicit risks of harm or pain). Since entrainment by tACS has also been shown to interact with task demands (Feurra et al., 2013), we may conclude it to be more effective in some contexts than in others. This has direct bearing on the potential applicability of tACS outside of the laboratory setting.

The same was already shown to be true for TMS (Silvanto and Pascual-Leone, 2008). Its effect depends critically on the level of ongoing activity within the targeted region (Romei et al., 2016): While in general low amplitude TMS enhances activity, whereas high amplitude TMS suppresses it, the shift from enhancement to suppression depends on the level of ongoing activity. Since ongoing activity depends globally on arousal and locally on task or situational demands, the same level of TMS can either enhance activity (when it targets low activity circuits) or suppress it (if it targets highly active circuits) (Silvanto and Cattaneo, 2017).

It stands to reason that similar logic would apply to tACS. However, tACS does not affect firing directly but rather shifts membrane potential. Therefore, it may bias cells toward firing or quiescence according to phase. Indeed, rather than modulating activity levels, tACS entrainment may affect the synchronization of oscillatory activity. This view is suggested by a dynamical systems framework—namely the theory of self-sustained oscillators (Pikovsky et al., 2003; Fröhlich, 2015)—in which entrainment is determined by the frequency of intrinsic oscillations, and its discrepancy with that of the external driving oscillation (frequency detuning). For a given level of detuning, there is a threshold value on the amplitude of rhythmic perturbation, above which entrainment is obtained. Thus, for a given level of oscillating driving force (sinusoidal AC injection) to be effective, a large enough pool of desynchronized “neural oscillators” in the viable frequency range (i.e., detuned within a level for which it is efficacious) must be available for entrainment. Accordingly, the lack of effect of tACS entrainment when eyes are shut can be explained by lack of oscillators available for entrainment within the requisite frequency band (as too many are already in synch). On average, stimulation in the individual alpha peak (IAF) is expected to be the most efficacious for a given level of perturbation, as it maximizes the number of oscillators within the viable detuning range. This, however, may not apply to individuals with wide alpha peaks.

From this perspective, experimental context can modulate the potential effect of tACS at a given frequency and current level, by changing the size of the potential pool of neural oscillators available for entrainment. For example, task specific alpha suppression can reduce the number of potential oscillators. On the other hand, reduced arousal can drive endogenously alpha oscillating networks to a highly synchronous state—thus limiting the pool of oscillators available for entrainment. Therefore, tasks that are highly engaging (thus ensuring high levels of arousal), yet do not involve local task induced alpha suppression would be ideally suited for tACS alpha entrainment.

In light of these considerations we chose to administer tACS during three background tasks that arguably differed in their induced degree of engagement (and thus arousal), and local task demands. To manipulate local task demand, we chose to utilize alongside the previously employed oddball task (Neuling et al., 2013; Helfrich et al., 2014a) (OddBl; not the most engaging of tasks), two engaging tasks of special interest, as they represent common pastime activities that patients could engage in while receiving tACS: listening to an audio book (AudBk), and free surfing on the web (FreeSrf). At the same while AudBk, like OddBl, should leave occipitoparietal networks unengaged, FreeSrf could be expected to induce widespread alpha suppression in these networks due to the high engagement with rich and rapidly changing visual stimuli characteristic of internet browsing. This should drastically curb the number of alpha oscillators available for entrainment.

In sum, AudBk might be the most efficient amongst the three tasks in setting the ground for entrainment, outside of temporal areas. In contrast, FreeSrf could be expected to engage many widespread networks, while maintaining relatively high levels of attention as compared to typical laboratory tasks, limiting the pool of local networks expressing alpha oscillations available for entrainment, and thus be the least effective of the three paradigms.

Second, the initial studies of alpha entrainment administered tACS using large rubber electrodes (5 × 7 cm Zaehle et al., 2010; Neuling et al., 2013; Helfrich et al., 2014a), with broad and rather diffuse effects. Arguably, for clinical applications it will become desirable to carefully target specific brain circuits. For example, focal control of alpha expression has been suggested to mediate selective attention and adaptive control (Sadaghiani and Kleinschmidt, 2016). Thus, perhaps focal modulation of alpha can be beneficial in target populations such as ADHD, or MCI (mild cognitive impairment) patients. There are studies suggesting that this should be possible using arrays of multiple smaller stimulating electrodes (Kronberg and Bikson, 2012; Helfrich et al., 2014b; Ruffini, 2015; Ruffini et al., 2017). However, the smaller the electrode surface is, the larger the current density. Hence the maximal non-painful current dosage reduces with electrode surface area. Therefore, it could be asked if this limitation could be detrimental to entrainment.

Another open question that remains is the extent to which entrainment is possible, as far as stimulation loci go: While alpha waves were originally thought to originate from thalamic pacemakers, later studies have shown that cortical networks can sustain alpha rhythms independently (Kristiansen and Courtois, 1949). And while at first it was conjectured that alpha originates from occipital regions, it has subsequently been shown to be generated in temporal and frontal regions as well (Cantero et al., 2002; Miller, 2007; Başar, 2012). Thus alpha generators seem to be present in all of cortex, a conclusion supported by modeling studies demonstrating that interplay between excitatory and inhibitory populations suffices to produce alpha oscillations (Jansen and Rit, 1995). Therefore, entrainment should be possible in most if not all cortex, provided strong enough current can be injected, and the local dynamics met by the induced electric fields are conducive to entrainment (i.e., a moderate level of ongoing alpha is expressed). However, several of the previous studies not only employed montages that explicitly targeted occipital/parietal areas, but the use of large stimulation electrodes precluded recording of activity directly under the area of stimulation. Therefore, another benefit that small electrodes could confer, is the ability to monitor entrainment aftereffects over widespread networks, especially so in the case of dual stimulating/recording electrodes, which can seek out small local effects that might result from various field inhomogeneity and shunting effects.

These last two considerations motivated us to explore alpha entrainment in a multi-electrode setting. We wanted to examine to what extent entrainment is possible—both in terms of the size imposed constraints on the amount of electric current as well as the extent in cortex to which it is possible to observe entrainment - using a stimulation montage of 8 relatively small (1 cm radius) dual recording/stimulating electrodes, arranged to mimic the montage proved effective in previous studies (Neuling et al., 2013; Helfrich et al., 2014a).

## Methods

### Participants

Seventy-eight healthy adults were recruited using the KU Leuven online registration system (*n* = 31, 24, and 23 for background tasks 1, 2, and 3, respectively, see below). Participants were screened for various medical indications such as epilepsy (see Supplementary Material 1: exclusion criteria). They were paid 15 euros per hour for participation. Out of the 78 participants, 8 were excluded before data were analyzed for having a stimulation sensation threshold smaller than a 100 μA, and an additional 8 for poor EEG signal quality (see Data analysis for rejection criteria), leaving a total of 62 (*n* = 22, 21, and 19 for background tasks 1, 2, and 3, respectively). Participant characteristics (after exclusion) are summarized in Table 1. Before proceeding, all participants signed an informed consent form. All experimental procedures were carried out in accordance with the Declaration of Helsinki and approved by the ethical committee of the faculty of Psychology and Educational Sciences of KU Leuven (SMEC). Each participant was assigned to one of six separate groups subsequently undergoing one of two stimulation types in the presence of one of three background tasks.

**Table 1:** Participant demographics: Number, sex, handedness, and age (mean and SEM) of participants for 3 background tasks and treatment type.

	**Audiobook**	**Free-surf**	**Oddball**	**Total**
SHAM	*n* = 9 (6M), 1LH, 26.6 ± 4.3 years	*n* = 8 (4M), 2LH, 21.1 ± 0.8 years	*n* = 9 (3M), 1LH, 21.2 ± 0.8 years	*n* = 26 (13M), 4LH, 23 ± 1.6 years
tACS	*n* = 13 (6M), 2LH, 22.6 ± 0.9 years	*n* = 13 (5M), 0LH, 22.1 ± 1 years	*n* = 10 (6M), 1LH, 23.5 ± 1.5 years	*n* = 36 (17M), 3LH, 22.7 ± 0.6 years
TOTAL	*n* = 22 (12F), 3LH, 24.2 ± 1.8 years	*n* = 21 (9F), 2LH, 21.7 ± 0.7 years	*n* = 19 (9F), 2LH, 22.4 ± 0.9 years	*n =* 62 (30F), 7LH, 22.8 ± 0.7 years

*M, male; LH, left-handed*.

### Electrophysiology setup

Stimulation and EEG recording were carried out with a StarStim stimulator (Neuroelectrics, Barcelona, Spain) using Ag/AgCl electrodes having the area of πcm (Fröhlich and McCormick, 2010) (PISTIM, Neuroelectrics, Barcelona, Spain). Electrodes were controlled with custom Matlab (Mathwoks, MA) code interfacing with the MatNIC package for controlling the StarStim (Neuroelectrics).

**Figure 1:**
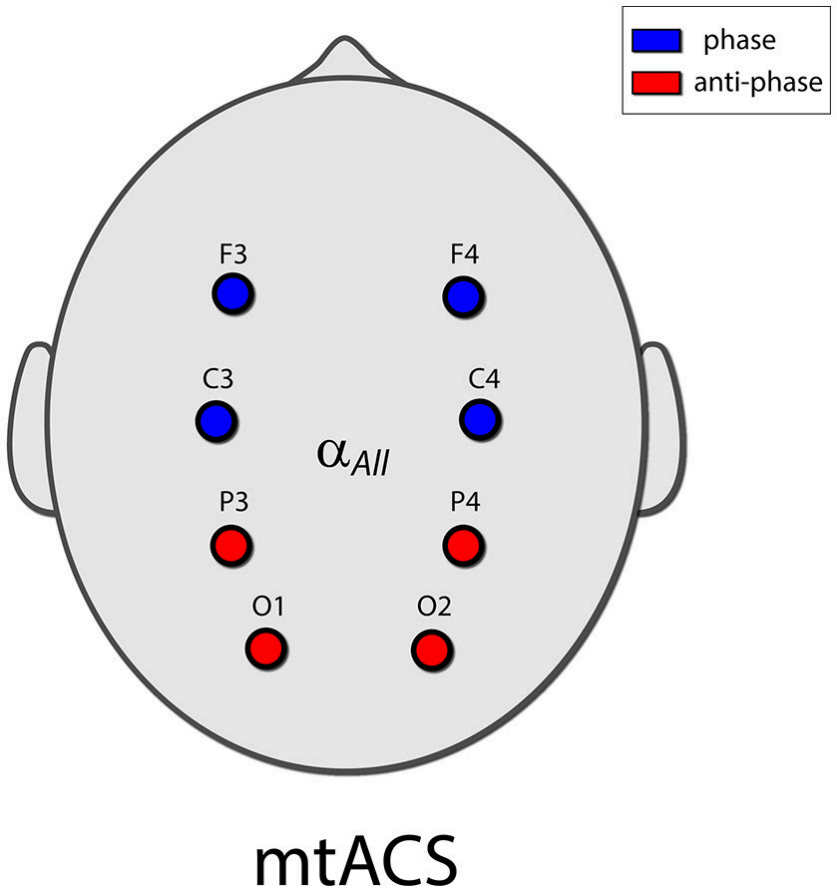
The mtACS paradigm. Stimulation frequency was determined as peak alpha for the spectrogram averaged over all electrodes. The anterior electrodes (C3,4 and F3,4) were phase locked, and in anti-phase to the posterior electrodes (O1,2 and P3,4). This in essence recreated the stimulation paradigm employed in (Neuling et al., 2013; Helfrich et al., 2014a) albeit in the multi-electrode scenario.

Eight electrodes were placed in a Neuroelectrics cap in positions F3, F4, C3, C4, P3, P4, O1, and O2 of the 10–20 International Electrode Placement System (Figure 1). Electrode gel was applied to keep impedance below 10 kΩ. DRL/CMS reference electrodes were clipped to the right earlobe. EEG was sampled at 500 Hz.

### Experimental procedure

Pre-stimulation baseline EEG was recorded for 5 min while participants were fixating on a white cross against a black background with their chin placed on a chin-rest (pre-EEG). This allowed semi-automatic extraction of peak alpha frequency, which determined frequency of stimulation (see below). Next, the sensation threshold was determined by a staircase procedure: initially current was ramped up by steps of 50 μA until threshold was exceeded then reduced by steps of 25 μA until sensation ceased. If necessary, current was increased again with the current step halved again, followed by decreasing the current in a minimal step of 10 μA, until a maximal level of current beneath threshold was found. Subsequent stimulation was administered at the selected level, or for participants who could not feel the stimulation at the maximal designated level of 400 μA (800 μA peak to peak).

The stimulation phase lasted 20 min in which tACS was delivered according to the stimulation parameters determined as described above. Current was ramped from 0 to the maximal amplitude in 10 s. In the sham condition current was ramped up as in the experimental condition but was immediately ramped down to zero, again in 10 s. Immediately before the end of the stimulation phase in the sham condition, current was ramped up to the maximal amplitude in 10 s. Stimulation was terminated by ramping down current in 10 s. This was immediately followed by 5 min of EEG recording (post-EEG) again with participants fixating on the cross with their chin placed on a chin-rest.

### mtACS stimulation

Electrodes were grouped into a posterior group (O1, O2, P3, P4) and an anterior group (C3, C4, F3, F4); the stimulating currents in the posterior and anterior groups were in anti-phase. Stimulation frequency was uniform and was determined by the peak alpha frequency of power averaged across all electrodes to facilitate peak detection.

### Background tasks

Stimulation was carried out in the presence of three different background tasks:

1. **AudBk**-During stimulation participants listened to a narration of “A people's history of the United States” by Howard Zinn read by Jeff Zinn, while EEG was recorded at rest (in silence). Passages containing rousing narrative or emotionally charged events were omitted.
2. **FreeSrf**-Participants were allowed to freely surf the web during stimulation, while again EEG was recorded at rest.
3. **OddBl**-Following (Neuling et al., 2013) participants carried out an auditory oddball task throughout both stimulation and EEG recording. In the oddball task, a 500 and a1,000 Hz tone (with 25% probability) were presented in random order for a 100 ms. Inter-stimulus intervals were randomly chosen to be either 8 or 12 s. Participants were asked to indicate the sound pitch by pressing either the L(ow) or H(igh) key on a standard keyboard.

### Data analysis

EEG data were preprocessed using BrainVisionAnalyzer 2 (Brain Products GmbH, Gilching, Germany). EEG was band-pass filtered with an order 2 Butterworth filter with 0.5–40 Hz bandwidth. Additionally, a 50 Hz notch filter was applied. We followed the standard procedure of artifact removal with ICA (Jung et al., 2000). For computing the unmixing matrix we selected a 200-s interval as the training dataset after skipping the initial 50 s of pre- and post-stimulation recordings. We visually inspected the obtained ICA components and removed those related to ocular and other types of artifacts. Next, we divided the continuous EEG into about 60 5 s consecutive segments. We used an automatic artifact rejection procedure for removal of the remaining artifacts due to large body movements, face/neck muscle activity, poor electrode contact, etc. Segments were discarded if the absolute voltage difference exceeded 50 μV between two neighboring sampling points and if the amplitude exceeded +100 or −100 μV within a segment. Only participants with at least 50% of retained segments were kept for the subsequent analysis (62 out of 70 subjects with a sensation threshold of at least 100 μA, for which on average over 90% of the segments were retained). We computed power spectra with the Welch method: each segment was padded by zeros to 4,096 samples, multiplied by a Hanning window and fast Fourier transformed. Then, segments were multiplied by their complex conjugate, and the absolute values were averaged. To compute relative alpha power, spectra were first log transformed for contrast. Next spectra were divided by the sum of power between 1 and 40 Hz. Power spectra in the alpha band (7.5–12.5 Hz) were extracted as a sum of the spectral lines, and also in the band defined for each individual by her intrinsic alpha frequency (IAF ± 2 Hz). Finally, the sum for the pre-EEG was subtracted from the sum for the post-EEG.

### Finite element modeling

Electric fields (EFs) were modeled using a finite element approach employing the ROAST toolbox (https://www.parralab.org/roast/). Field values were obtained from the maximal phase—that is assuming the average injected peak current value. Modeling was done using a standard head template. To assess EF level under a given recording electrode (e.g., O1) EF was averaged in all voxels within a radius of 1 cm from electrode position.

### Long range temporal correlations

To compute alpha LRTC (Peng et al., 1994; Linkenkaer-Hansen et al., 2001; Hardstone et al., 2012) data were filtered into the α and IAF ± 2 Hz bands. This was followed by applying the Hilbert transform to the filtered signals to find signal envelope. Next, DFA (detrended fluctuation analysis) was applied to each channel. Finally, we derived $\alpha =\frac{\alpha -\alpha_{0}}{\alpha_{0}}$ where α denotes the DFA exponent (which indicates the extent of long range correlations) after stimulation and α_0_ baseline DFA exponent seen with a given electrode.

### Coherence analysis

Following (Neuling et al., 2013), we computed the coherence between L/R electrode pairs (e.g., O1/O2). The coherence for a given frequency is given by $C_{xy}\left(f\right)=\frac{|P_{xy}\left(f\right){|}^{2}}{P_{xx}\left(f\right)P_{yy}\left(f\right)}$. *C*_*xy*_was computed for all frequencies either in the alpha band or IAF ± 2 Hz and integrated (summed) both for pre and post EEG to obtain *C*_*xy*_(α) and *C*_*xy*_(α_0_) respectively. Finally, we derived $\Delta C_{xy}(\alpha )=\frac{C_{xy}(\alpha )-C_{xy}(\alpha_{0})}{C_{xy}(\alpha_{0})}$ for all four electrode pairs.

## Results

To exclude confounds resulting from the demographic characteristics of participants (Table 1), a 3 × 2 (Background Task × Stimulation Type) ANOVA or factorial logistic regression (on categorical variables) was carried out on age, handedness and sex. No significant differences were found.

**Figure 2:**
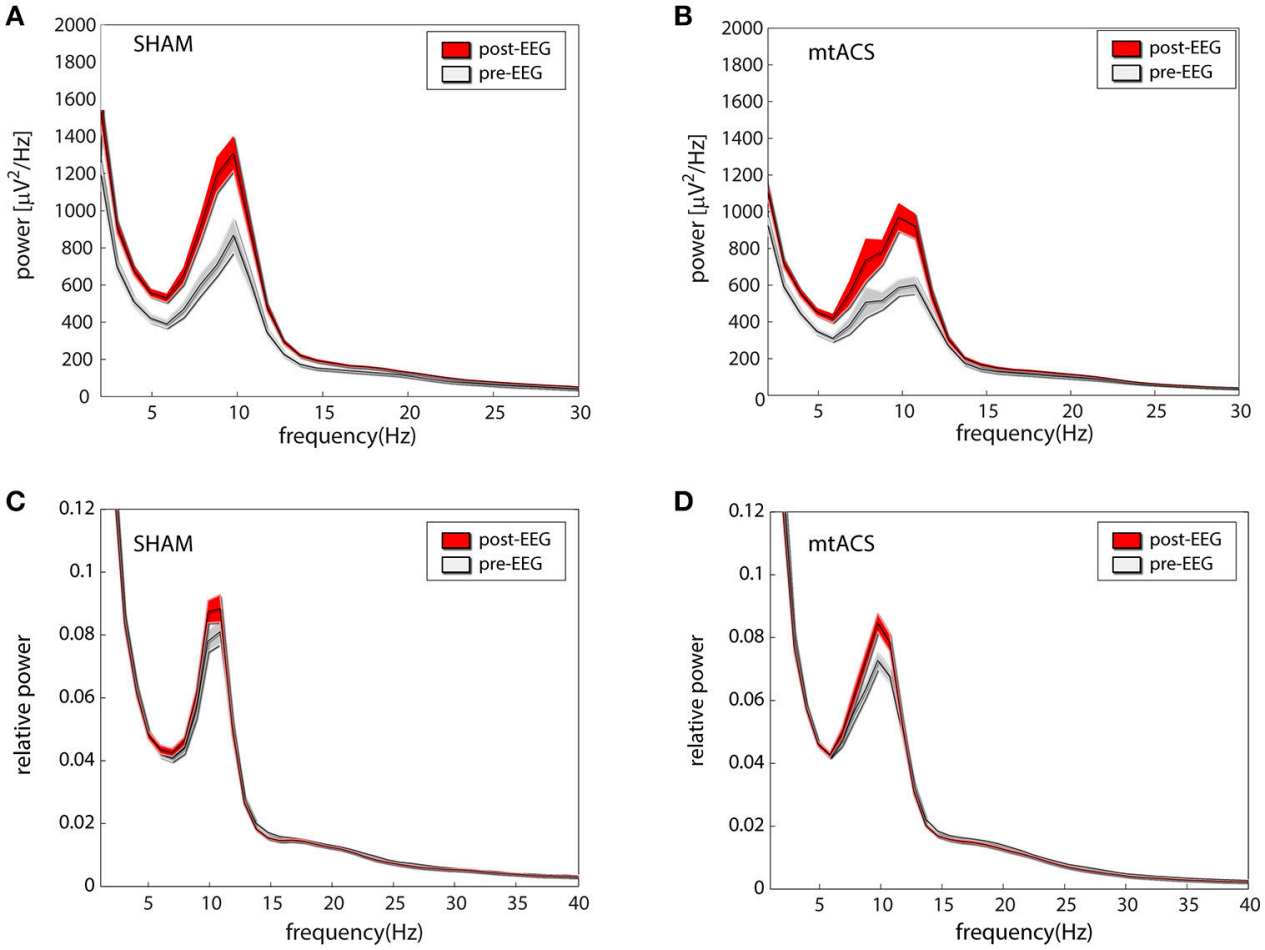
Effects of stimulation. **(A,B)** The average (across participants and electrodes) spectrograms before (gray) and after (red) stimulation for mtACS stimulation and sham. **(C,D)** Same as the above after normalizing spectra to power in the 1–40 Hz band. Envelope represents SE.

Figures 2A,B portrays the average EEG power before stimulation (pre-EEG) and afterwards (post-EEG). Statistical analyses were carried out on relative power in the alpha band (see section Methods). An ANOVA with factors Background Task (3 levels), Stimulation Type (2 levels), and Electrode Position (4 levels- O,P,C and F) was applied to the pre/post difference of the relative alpha power. We did not find effects for stimulation [*F*_(1, 472)_ = 0.7, *p* > 0.35], background task [*F*_(2, 472)_ = 1.23, *p* > 0.25], and electrode position [F_(3, 472)_ = 1.19, *p* > 0.3]. Similarly, none of the interactions between conditions were found to be significant, triple interaction included. Relative power before and after stimulation is portrayed in Figures 2C,D for both stimulation conditions.

The analysis was repeated using the average IAF power—the relative power in the ±2 Hz band around individual alpha peak frequencies. Again, we did not find effects of stimulation [*F*_(1, 472)_ = 1.61, *p* > 0.2], background task [*F*_(2, 472)_ = 0.17, *p* > 0.8], nor electrode position [*F*_(3, 472)_ = 0.87, *p* > 0.4]. Similarly, none of the interactions between conditions were found to be significant, triple interaction included.

It could be argued that standard stimulation was not efficacious due to the weaker levels of current injected as compared to Neuling et al. (2013). However, current level *per se* was not the direct cause of entrainment failing: although an average stimulation magnitude of ~877 μA was used there, and on average only 272 ± 18 μA in our experiment, given that we used 8 electrodes the total injected current was in fact four times larger in our study, that is 1,089 ± 72 μA.

**Figure 3:**
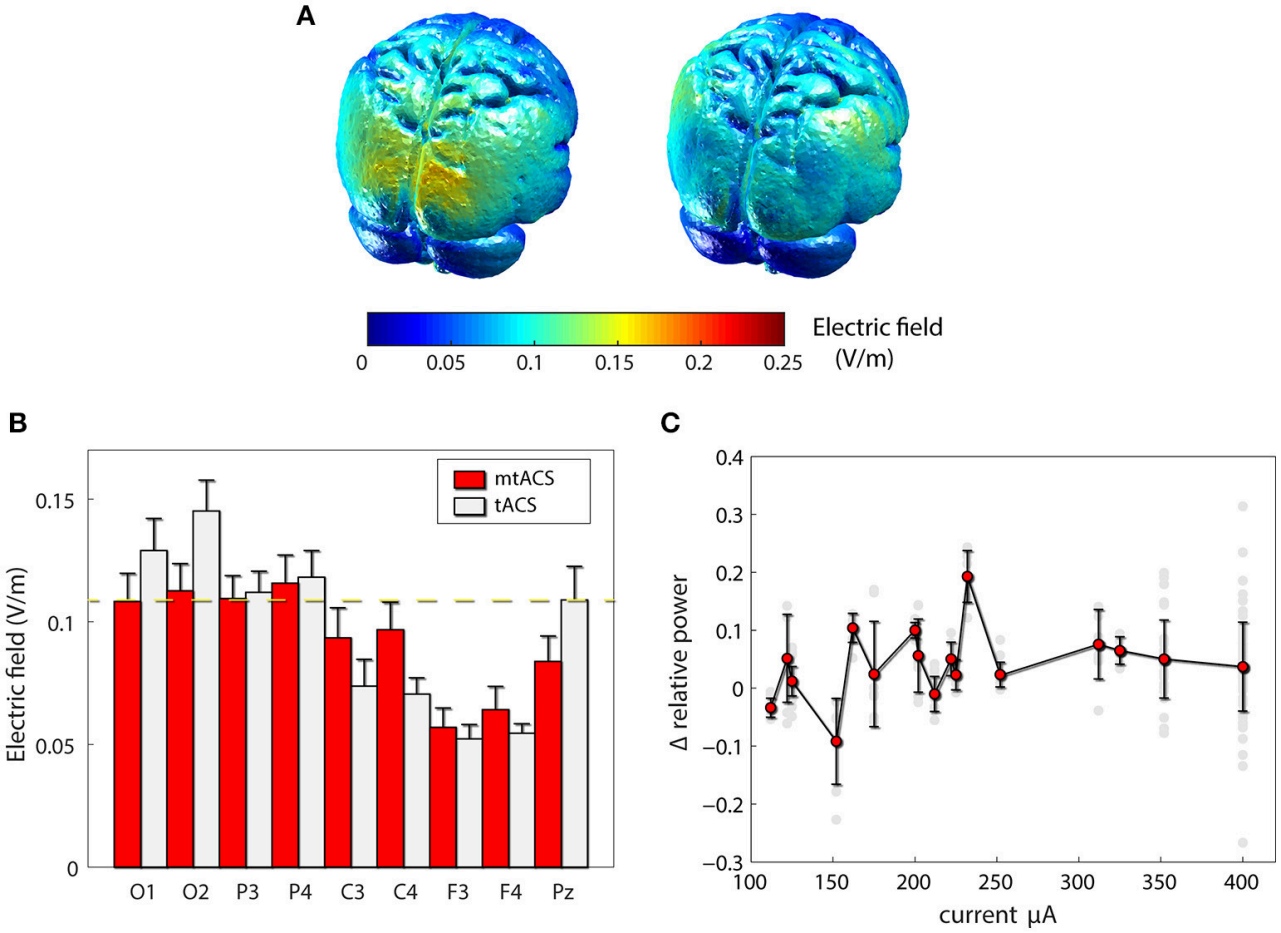
Effects of stimulation. **(A)** Finite element modeling of induced electrical field (EF). Left: EF for the paradigm of Neuling et al(Neuling et al., 2013). Right: the result for our mtACS paradigm. **(B)** the average field strength (mean ± SD) under the recording electrodes in both studies. **(C)** Alpha modulation as a function of injected current. (gray – individual electrodes, red – mean ± SD modulation (across electrodes and subjects) per current level).

To rule out the possibility that our failure to replicate the previous findings was due to inefficiency of our mtACS montage, we carried out finite element modeling of induced electrical fields (Figure 3A) in both the original and our own paradigm. Model-predicted EF levels correlated highly with human invasive electrophysiology measurement (Huang et al., 2017) and yielded EF values commensurate with our simulation results. While indeed, Neuling et al. (2013) induced a stronger field in occipital areas, around Pz (the electrode they used to estimate power), field strength was comparable to that of our posterior and parietal recording sites (see Figure 3B). Furthermore, in subjects who were administered higher currents (>250 μA), nevertheless no correlation whatsoever was found between stimulation amplitude and alpha modulation, neither across all electrodes (*r* = −0.01, *p* > 0.85, *df* = 150; Figure 3C), nor for occipital electrodes alone (*r* = −0.09, *p* > 0.55, *df* = 36).

**Figure 4:**
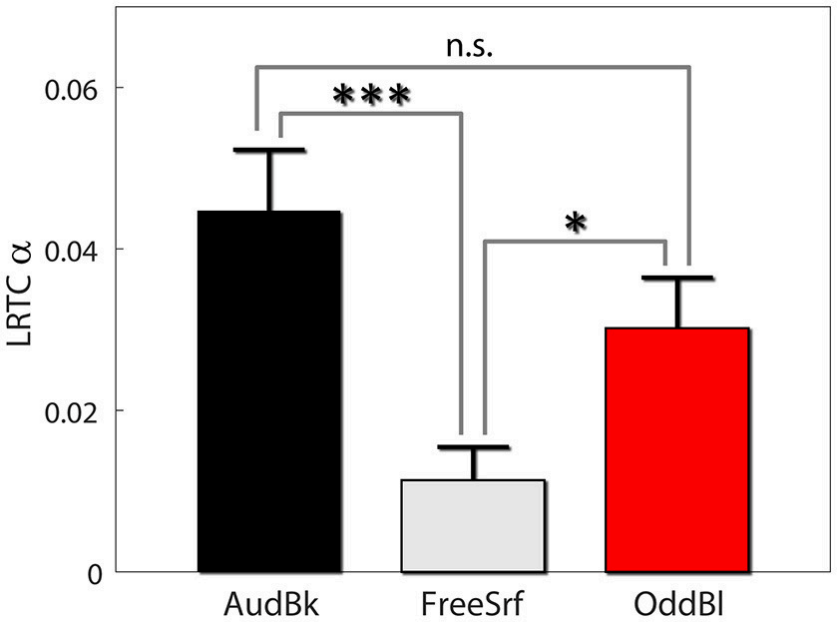
Alpha LRTC aftereffects. Results are presented as mean ± SE. *Post-hoc p*-values were FDR corrected. ^*^p < 0.05, ^***^p < 0.001.

We also carried out both long range temporal correlation (LRTC) and coherence analysis in the alpha band to seek for aftereffects (see section Methods). For both measures we derived the difference between pre and post EEG expression level divided by the basal level: $\alpha =\frac{\alpha -\alpha_{0}}{\alpha_{0}}$. First, we found that alpha LRTC differed between background tasks [*F*_(2, 472)_ = 7.53, *p* < 0.001], but not for stimulation type [*F*_(1, 472)_ = 0.23, *p* > 0.6], nor electrode position [*F*_(3, 472)_ = 1.4, *p* > 0.2]. None of the interactions were significant. *Post hoc* analysis indicated that alpha LRTC were significantly lower for the free surfing condition as compared both to the audiobook and oddball conditions (*p* < 0.001, *p* < 0.05, *p* > 0.15 FDR corrected with df 342, 326, and 318 respectively; see Figure 4). Similar results were obtained for IAF ± 2 Hz signals.

We did not find any residual changes in coherence for stimulation [*F*_(1, 224)_ = 0.37, *p* > 0.55), background task [*F*_(2, 224)_ = 1.8, *p* > 0.15], electrode position [*F*_(3, 224)_ = 1.14, *p* > 0.3], nor any interactions. Similar results were obtained for IAF ± 2 Hz signals.

## Discussion

We employed tACS stimulation with 8 electrodes in the presence of three background tasks. Our mtACS paradigm failed to replicate previous results of tACS alpha entrainment (Neuling et al., 2013; Helfrich et al., 2014a): using a multi-electrode (8 electrodes) stimulation setup, inducing a posterior/anterior bidirectional current flow in matched intrinsic alpha peak stimulation frequency, we did not observe the expected increase in alpha oscillations after stimulation relative to the sham condition.

This outcome could not be explained by injected current levels—total injected current levels in our mtACS paradigm exceeded those sufficient for entrainment in the original study. While it had, according to finite element modeling, higher electric field values in superficial occipital areas compared to our study, this was not the case in their actual recording site. Further still, modulation of alpha did not scale with injected current amplitude, casting doubt if the lack of findings can simply be explained by EF levels. Indeed, for both paradigms simulations suggest field levels much smaller than the minimal levels found to be efficacious in animals (Reato et al., 2013), or expected to be efficacious in humans (Vöröslakos et al., 2018).

Given that we employed dual stimulating/recording electrodes, we could not record EEG alongside stimulation, but only preceding and following stimulation, and therefore could not directly verify whether our manipulation of background task indeed resulted in modulation of basal level alpha activity. However, we found a significant aftereffect for alpha LRTC for background task, but not for stimulation conditions. This indicates that the task manipulation was successful in modulating alpha.

Our results suggest that viability of alpha entrainment employing arrays of multiple electrodes, at least where small and spatially separated electrodes are concerned, would benefit from direct corroboration with invasive electrophysiology [e.g., in epileptic patients with implants Huang et al., 2017]. And in general, additional studies would help understand to what extent tACS alpha entrainment is a robust phenomenon.

## Author contributions

TF, AN, and CvL designed the study. TF and AZ conducted the experiments, which were supervised by FD. TF prepared the figures and conducted the analyses. TF, AN, CvL, AZ, and FD wrote the manuscript.

### Conflict of interest statement

The authors declare that the research was conducted in the absence of any commercial or financial relationships that could be construed as a potential conflict of interest.

## References

[B1] Başar E. (2012). A review of alpha activity in integrative brain function: fundamental physiology, sensory coding, cognition and pathology. Int. J. Psychophysiol. 86, 1–24. 10.1016/j.ijpsycho.2012.07.002 22820267

[B2] Cantero J. L. Atienza M. Salas R. M. (2002). Human alpha oscillations in wakefulness, drowsiness period, and REM sleep: different electroencephalographic phenomena within the alpha band. Neurophysiol. Clin. Neurophysiol. 32, 54–71. 10.1016/S0987-7053(01)00289-1 11915486

[B3] Feurra M. Pasqualetti P. Bianco G. Santarnecchi E. Rossi A. Rossi S. (2013). State-dependent effects of transcranial oscillatory currents on the motor system: what you think matters. J. Neurosci. 33, 17483–17489. 10.1523/JNEUROSCI.1414-13.2013 24174681 PMC6618358

[B4] Fröhlich F. (2015). Experiments and models of cortical oscillations as a target for noninvasive brain stimulation. Prog. Brain Res. 222, 41–73. 10.1016/bs.pbr.2015.07.025 26541376

[B5] Fröhlich F. McCormick D. A. (2010). Endogenous electric fields may guide neocortical network activity. Neuron 67, 129–143. 10.1016/j.neuron.2010.06.005 20624597 PMC3139922

[B6] Hardstone R. Poil S. S. Schiavone G. Jansen R. Nikulin V. V. Mansvelder H. D.. (2012). Detrended fluctuation analysis: a scale-free view on neuronal oscillations. Front. Physiol. 3:450. 10.3389/fphys.2012.00450 23226132 PMC3510427

[B7] Helfrich R. F. Knepper H. Nolte G. Strüber D. Rach S. Herrmann C. S.. (2014b). Selective modulation of interhemispheric functional connectivity by HD-tACS shapes perception. PLoS Biol. 12, e1002031. 10.1371/journal.pbio.1002031 25549264 PMC4280108

[B8] Helfrich R. F. Schneider T. R. Rach S. Trautmann-Lengsfeld S. A. Engel A. K. Herrmann C. S. (2014a). Entrainment of brain oscillations by transcranial alternating current stimulation. Curr. Biol. 24, 333–339. 10.1016/j.cub.2013.12.041 24461998

[B9] Huang Y. Liu A. A. Lafon B. Friedman D. Dayan M. Wang X.. (2017). Measurements and models of electric fields in the *in vivo* human brain during transcranial electric stimulation. Elife 6:e18834. 10.7554/eLife.18834 28169833 PMC5370189

[B10] Jansen B. H. Rit V. G. (1995). Electroencephalogram and visual evoked potential generation in a mathematical model of coupled cortical columns. Biol. Cybern. 73, 357–366. 10.1007/BF00199471 7578475

[B11] Jung T. P. Makeig S. Humphries C. Lee T. W. McKeown M. J. Iragui V.. (2000). Removing electroencephalographic artifacts by blind source separation. Psychophysiology 37, 163–178. 10.1111/1469-8986.3720163 10731767

[B12] Kristiansen K. Courtois G. (1949). Rhythmic electrical activity from isolated cerebral cortex. Electroencephal. Clin. Neurophysiol. 1, 265–272. 10.1016/0013-4694(49)90191-1 18135418

[B13] Kronberg G. Bikson M. (2012). Electrode assembly design for transcranial direct current stimulation: a FEM modeling study, in 2012 Annual International Conference of the IEEE Engineering in Medicine and Biology Society (EMBC) (IEEE), 891–895. 10.1109/EMBC.2012.6346075 23366036

[B14] Linkenkaer-Hansen K. Nikouline V. V. Palva J. M. Ilmoniemi R. J. (2001). Long-range temporal correlations and scaling behavior in human brain oscillations. J. Neurosci. 21, 1370–1377. 10.1523/JNEUROSCI.21-04-01370.2001 11160408 PMC6762238

[B15] Miller R. (2007). Theory of the normal waking EEG: from single neurones to waveforms in the alpha, beta and gamma frequency ranges. Int. J. Psychophysiol. 64, 18–23. 10.1016/j.ijpsycho.2006.07.009 16997407

[B16] Neuling T. Rach S. Herrmann C. S. (2013). Orchestrating neuronal networks: sustained after-effects of transcranial alternating current stimulation depend upon brain states. Front. Hum. Neurosci. 7:161. 10.3389/fnhum.2013.00161 23641206 PMC3639376

[B17] Peng C.-K. Buldyrev S. V. Havlin S. Simons M. Stanley H. E. Goldberger A. L. (1994). Mosaic organization of DNA nucleotides. Phys. Rev. 49:1685. 10.1103/PhysRevE.49.1685 9961383

[B18] Pikovsky A. Rosenblum M. Kurths J. (2003). Synchronization: A Universal Concept in Nonlinear Sciences, Vol. 12 Cambridge, UK: Cambridge University Press.

[B19] Reato D. Rahman A. Bikson M. Parra L. C. (2013). Effects of weak transcranial alternating current stimulation on brain activity—a review of known mechanisms from animal studies. Front. Hum. Neurosci. 7:687. 10.3389/fnhum.2013.00687 24167483 PMC3805939

[B20] Romei V. Gross J. Thut G. (2010). On the role of prestimulus alpha rhythms over occipito-parietal areas in visual input regulation: correlation or causation? J. Neurosci. 30, 8692–8697. 10.1523/JNEUROSCI.0160-10.2010 20573914 PMC6634639

[B21] Romei V. Thut G. Silvanto J. (2016). Information-based approaches of noninvasive transcranial brain stimulation. Trends Neurosci. 39, 782–795. 10.1016/j.tins.2016.09.001 27697295

[B22] Ruffini G. (2015). Application of the reciprocity theorem to EEG inversion and optimization of EEG-driven transcranial current stimulation (tCS, including tDCS, tACS, tRNS). arXiv preprint arXiv:1506.04835.

[B23] Ruffini G. Martinez-Ruiz de Lara C. Martínez-Zalacaín I. Cardoner N. (2017). Optimized multielectrode tDCS modulates corticolimbic networks. Brain Stimul. 10:e14 10.1016/j.brs.2016.11.064

[B24] Ruhnau P. Neuling T. Fusc,á M. Herrmann C. S. Demarchi G. Weisz N. (2016). Eyes wide shut: transcranial alternating current stimulation drives alpha rhythm in a state dependent manner. Sci. Reports 6:27138. 10.1038/srep27138 27252047 PMC4890046

[B25] Sadaghiani S. Kleinschmidt A. (2016). Brain Networks and α-Oscillations: structural and functional foundations of cognitive control. Trends Cogn. Sci. 20, 805–817. 10.1016/j.tics.2016.09.004 27707588

[B26] Silvanto J. Cattaneo Z. (2017). Common framework for “virtual lesion” and state-dependent TMS: the facilitatory/suppressive range model of online TMS effects on behavior. Brain Cogn. 119, 32–38. 10.1016/j.bandc.2017.09.007 28963993 PMC5652969

[B27] Silvanto J. Pascual-Leone A. (2008). State-dependency of transcranial magnetic stimulation. Brain Topogr. 21:1. 10.1007/s10548-008-0067-0 18791818 PMC3049188

[B28] Vöröslakos M. Takeuchi Y. Brinyiczki K. Zombori T. Oliva A. Fernández-Ruiz A.. (2018). Direct effects of transcranial electric stimulation on brain circuits in rats and humans. Nat. Commun. 9:483. 10.1038/s41467-018-02928-3 29396478 PMC5797140

[B29] Zaehle T. Rach S. Herrmann C. S. (2010). Transcranial alternating current stimulation enhances individual alpha activity in human EEG. PLoS ONE 5:e13766. 10.1371/journal.pone.0013766 21072168 PMC2967471

